# Redox-Epigenetic Crosstalk in Plant Stress Responses: The Roles of Reactive Oxygen and Nitrogen Species in Modulating Chromatin Dynamics

**DOI:** 10.3390/ijms26157167

**Published:** 2025-07-24

**Authors:** Cengiz Kaya, Ioannis-Dimosthenis S. Adamakis

**Affiliations:** 1 Soil Science and Plant Nutrition Department, Harran University, Sanliurfa 63200, Turkey; ckaya@harran.edu.tr; 2 Section of Botany, Department of Biology, National and Kapodistrian University of Athens, 15784 Athens, Greece

**Keywords:** chromatin dynamics, crop improvement, epigenomic analysis, gene regulation, molecular signaling, stress adaptation

## Abstract

Plants are constantly exposed to environmental stressors such as drought, salinity, and extreme temperatures, which threaten their growth and productivity. To counter these challenges, they employ complex molecular defense systems, including epigenetic modifications that regulate gene expression without altering the underlying DNA sequence. This review comprehensively examines the emerging roles of reactive oxygen species (ROS) and reactive nitrogen species (RNS) as central signaling molecules orchestrating epigenetic changes in response to abiotic stress. In addition, biotic factors such as pathogen infection and microbial interactions are considered for their ability to trigger ROS/RNS generation and epigenetic remodeling. It explores how ROS and RNS influence DNA methylation, histone modifications, and small RNA pathways, thereby modulating chromatin structure and stress-responsive gene expression. Mechanistic insights into redox-mediated regulation of DNA methyltransferases, histone acetyltransferases, and microRNA expression are discussed in the context of plant stress resilience. The review also highlights cutting-edge epigenomic technologies such as whole-genome bisulfite sequencing (WGBS), chromatin immunoprecipitation sequencing (ChIP-seq), and small RNA sequencing, which are enabling precise mapping of stress-induced epigenetic landscapes. By integrating redox biology with epigenetics, this work provides a novel framework for engineering climate-resilient crops through the targeted manipulation of stress-responsive epigenomic signatures.

## 1. Introduction

Plants are constantly exposed to a range of abiotic stressors, including drought, salinity, extreme temperatures, and heavy metal toxicity, that significantly impair their growth, development, and productivity [1,2]. These stress conditions trigger intricate physiological, biochemical, and molecular responses aimed at maintaining homeostasis and ensuring survival. Central to these responses is the regulation of gene expression through epigenetic mechanisms, which allow for flexible and reversible modifications in gene activity without altering the underlying DNA sequence. Epigenetic modifications encompass DNA methylation, histone post-translational modifications, and small RNA-mediated gene regulation, and are vital in facilitating rapid and adaptive stress responses [3,4].

Recent studies have underscored the dual role of ROS and RNS not only as cytotoxic agents under excessive accumulation but also as critical signaling molecules involved in stress perception and signal transduction [5,6]. Under controlled concentrations, ROS, such as superoxide radicals (O_2_^•−^), hydrogen peroxide (H_2_O_2_), and hydroxyl radicals (•OH), and RNS, primarily nitric oxide (NO) and peroxynitrite (ONOO^−^), regulate a wide range of physiological processes by modulating key signaling pathways and interacting with epigenetic machinery [7,8].

ROS and RNS influence gene regulation by altering the activity of DNA methyltransferases (DNMTs), histone acetyltransferases (HATs), histone deacetylases (HDACs), and components of the small RNA biosynthesis pathways. For example, ROS can induce oxidative modifications that affect chromatin structure and transcription factor binding, while NO-mediated S-nitrosylation of epigenetic enzymes can influence DNA methylation dynamics and histone acetylation status [9,10,11]. Furthermore, both ROS and RNS have been implicated in regulating small RNA pathways, thereby influencing post-transcriptional gene silencing mechanisms essential for stress adaptation [12,13].

With the advent of high-throughput epigenomic technologies, such as whole-genome bisulfite sequencing (WGBS) and chromatin immunoprecipitation sequencing (ChIP-seq), scientists can now precisely map stress-induced epigenetic changes at a genome-wide scale [14,15]. These tools have shed light on how ROS and RNS orchestrate chromatin modifications and transcriptional reprogramming during environmental stress responses. In addition, ATAC-seq (Assay for Transposase-Accessible Chromatin using sequencing) is increasingly applied to investigate genome-wide changes in chromatin accessibility under abiotic stress conditions. Studies in apple and Tritipyrum have demonstrated that drought and salt stress, respectively, trigger dynamic chromatin remodeling associated with transcriptional reprogramming [16,17]. Enzyme-tethering techniques such as CUT&Tag (Cleavage Under Targets and Tagmentation) have further advanced histone modification profiling in plants, offering higher resolution, reduced background noise, and lower input requirements compared to traditional ChIP-seq [18,19].

While recent reviews such as Shriti et al. [20] and Ramakrishnan et al. [4] have significantly advanced our understanding of the interplay between redox signaling and epigenetic regulation under abiotic stress, they often depict redox dynamics and epigenetic mechanisms as parallel, loosely coordinated systems. In contrast, our review emphasizes a more integrated and co-evolved framework in which reactive oxygen and nitrogen species (ROS/RNS) are not only modulators but also core components of the epigenetic machinery itself. Drawing on the synthesis presented by Tresas et al. [21], we highlight that redox signals influence all major layers of epigenetic control, including DNA methylation, histone modifications, and small RNA pathways, through enzyme regulation, chromatin remodeling, and transcriptional reprogramming.

For instance, the DNA demethylase ROS1, essential for stress adaptation and gene regulation, functions as a redox-sensitive Fe–S cluster enzyme; its activity depends on cellular redox status, linking ROS levels to active DNA demethylation and epigenetic homeostasis [22,23]. Similarly, histone modifiers, namely Suppressor of Variegation 3–9 Homologs 4, 5, and 6 (SUVH4/5/6), which install and maintain H3K9me2 marks associated with transposon silencing and stress adaptation, and Polycomb Repressive Complex 2 (PRC2) members, which catalyze H3K27me3, contribute to dynamic chromatin reprogramming in response to environmental stimuli, thereby tightly regulating stress-responsive gene expression [24]. Moreover, GCN5 plays a dual role in microRNA biogenesis, positively regulating stress-inducible MIRNA gene expression while indirectly repressing miRNA processing components such as *DCL1*, *SE*, *HYL1*, and *AGO1* via histone acetylation dynamics [25]. As reviewed by Tresas et al. [21], chromatin rearrangements under abiotic and biotic stress—including nucleosome repositioning, histone acetylation, and demethylation—are central to stress-responsive gene regulation and epigenetic memory in plants.

Thus, rather than viewing ROS/RNS and epigenetic regulation as independent or sequential layers, our model posits that redox molecules act as central, dynamic hubs that co-regulate epigenetic architecture via enzymatic, structural, and transcriptional modifications. This unified perspective offers a mechanistic foundation for developing stress-resilient crops by targeting redox-epigenetic feedback loops that are already deeply embedded in the plant’s adaptive repertoire.

## 2. ROS and RNS Signaling in Plant Stress Responses

Reactive oxygen species and reactive nitrogen species are pivotal signaling molecules in plant responses to environmental stress. Although traditionally associated with oxidative and nitrosative damage, their spatially and temporally regulated production initiates complex signaling networks that mediate stress perception, transduction, and adaptive gene expression programs [6,7]. These molecules regulate redox homeostasis, mediate crosstalk with phytohormones, and interface with epigenetic regulatory mechanisms, making them central to the modulation of stress-responsive pathways [26].

### 2.1. ROS Signaling Pathways

ROS, including O_2_^•−^, H_2_O_2_, and •OH, are primarily generated in chloroplasts, mitochondria, and peroxisomes as metabolic byproducts. Under stress conditions, ROS levels rise, serving both as damaging agents and as key secondary messengers in signaling cascades that activate defense genes [27].

Under stress, ROS accumulation can trigger oxidative damage to nucleic acids, lipids, and proteins, impairing cellular integrity. However, at controlled levels, ROS play pivotal roles in cell signaling and adaptive responses by modulating the expression of stress-responsive genes and proteins. This dual role of ROS highlights their function as “double-edged swords” in plant stress biology [28,29].

Key enzymes involved in ROS homeostasis include NADPH oxidases (NOXs), superoxide dismutases (SODs), catalases, and various peroxidases. NADPH oxidases catalyze the production of O_2_^•−^ from oxygen, while SOD enzymes dismutate O_2_^•−^ into H_2_O_2_, which is further detoxified by catalases and peroxidases [30]. This enzymatic machinery ensures a fine balance between ROS production and scavenging, enabling ROS to function as signaling molecules without inducing cytotoxicity [7].

Hydrogen peroxide, in particular, acts as a diffusible secondary messenger in numerous signaling pathways. It modulates processes such as programmed cell death, stomatal closure, and systemic-acquired resistance by interacting with specific target proteins and transcription factors [31]. For instance, H_2_O_2_ is known to activate Mitogen-Activated Protein Kinase 6 (MAPK6), which phosphorylates the nitrate reductase isoform NIA2, enhancing its activity and promoting NO synthesis. This H_2_O_2_-induced NO production plays a critical role in lateral root development by modulating root growth and signaling pathways [32]. MAPKs such as MPK3 and MPK6 play critical roles in regulating the expression of defense-related genes and in coordinating crosstalk between ROS and phytohormone signaling networks [33].

In addition to antioxidant enzymes, plants employ sophisticated repair mechanisms to mitigate ROS-induced damage and preserve cellular integrity. Oxidative stress can cause DNA lesions such as 8-oxoguanine, which are corrected through base excision repair (BER) pathways involving key enzymes like 8-oxoguanine glycosylase (OGG1) and formamidopyrimidine-DNA glycosylase (FPG) [34,35,36]. These DNA repair proteins excise oxidized bases and initiate a repair cascade critical for genome stability under stress. Beyond DNA, ROS also damage proteins and lipids, prompting the activation of methionine sulfoxide reductases (MsrA/B) that restore oxidized methionine residues [37,38], as well as lipid repair enzymes that counteract peroxidation products like 4-hydroxy-2-nonenal (4-HNE) [39,40].

ROS-mediated signaling is further intertwined with calcium (Ca^2+^) signaling and phytohormones such as abscisic acid (ABA), salicylic acid (SA), and jasmonic acid (JA). For example, a study indicates that Ca^2+^ wave propagation in plants involves a ROS-assisted, calcium-induced, calcium-release mechanism, where ROS production by NADPH oxidase (AtRBOHD) and Ca^2+^ release via the vacuolar ion channel TPC1 work in tandem. This mechanism enables systemic signaling by coordinating rapid Ca^2+^ and ROS waves, with disruptions to either component, through ROS scavengers, NADPH oxidase inhibitors, or TPC1 dysfunction, resulting in reduced wave transmission speeds [41]. Similarly, ROS and ABA interact to regulate stomatal closure during drought stress, with ROS produced by RBOHD and RBOHF playing a key role in guard cell signaling. ABA-induced ROS accumulation exhibits specific spatial and temporal patterns, requiring balanced ROS production and antioxidant activity to maintain ROS homeostasis and ensure effective stress responses [42].

### 2.2. RNS Signaling Pathways

Reactive nitrogen species, primarily NO and ONOO^−^, are pivotal components of plant stress signaling networks. These molecules are produced as part of normal cellular metabolism and are known to accumulate rapidly under abiotic and biotic stress conditions. NO, in particular, has emerged as a central signaling molecule regulating diverse physiological and molecular processes in plants, including antioxidant defense, ion homeostasis, and post-translational modifications [43].

NO is synthesized through two primary pathways in plants: the nitrate reductase (NR)-dependent pathway and the nitric oxide synthase (NOS)-like pathway. Nitrate reductase catalyzes the reduction in nitrite (NO_2_^−^) to NO in the cytoplasm, while NOS-like enzymes, though less characterized in plants compared to animals, contribute to NO generation under specific conditions [44]. The dynamic regulation of NO synthesis allows plants to fine-tune its levels to meet the demands of stress signaling without triggering nitrosative damage.

One of NO’s critical roles under stress is its involvement in post-translational modifications, including S-nitrosylation and tyrosine nitration. S-nitrosylation, the covalent attachment of an NO moiety to cysteine residues in proteins, alters protein structure, activity, and interactions, thereby modulating stress-responsive pathways [45]. Similarly, tyrosine nitration, the addition of a nitro group to tyrosine residues via reactive nitrogen species like peroxynitrite, modifies protein function, stability, and signaling interactions [46]. These modifications serve as key mechanisms by which NO enhances stress tolerance and orchestrates cellular responses to environmental challenges.

Excessive RNS accumulation during prolonged stress can result in nitrosative stress, characterized by the disruption of cellular homeostasis and oxidative damage. However, NO also functions as a regulator of redox balance by interacting with ROS and phytohormones. This crosstalk between RNS, ROS, and phytohormonal signaling pathways facilitates the activation of adaptive responses. For instance, the interplay between NO and H_2_O_2_ has been shown to amplify stress signaling through coordinated activation of MAPKs and transcription factors [47].

In addition to modulating protein function, NO influences gene expression under stress conditions by modifying transcription factors and signaling proteins. NO-driven modifications of transcription factors and signaling proteins directly influence gene expression under stress conditions [48].

The integration of NO signaling with other molecular networks underscores its multifaceted role in plant stress adaptation. For example, NO regulates stomatal closure during drought stress by modulating Ca^2+^ signaling and ABA sensitivity [49]. Additionally, NO-mediated signaling contributes to salinity tolerance by enhancing ion homeostasis and mitigating sodium toxicity [50].

### 2.3. Crosstalk Between ROS and RNS Signaling

The signaling pathways of ROS and RNS are intricately interconnected, forming a dynamic redox signaling network that plays a crucial role in the regulation of stress responses in plants. Both ROS and RNS are key mediators of cellular homeostasis and stress adaptation, yet their interactions can significantly influence the magnitude and nature of the stress response.

A central aspect of ROS and RNS crosstalk involves the formation of ONOO^−^, a highly reactive compound that forms when NO reacts with O_2_^•−^. Peroxynitrite is a potent oxidant and nitrating agent that can exacerbate oxidative and nitrosative stress, leading to cellular damage through the modification of proteins, lipids, and nucleic acids [51]. The accumulation of ONOO^−^ under stress conditions can further amplify stress signaling and contribute to the activation of stress-related genes involved in defense responses.

Interestingly, NO can also function as a scavenger of ROS, particularly O_2_^•−^ and •OH, thus contributing to the restoration of redox homeostasis and preventing oxidative damage. In this capacity, NO serves as an antioxidant, providing a protective role against oxidative stress by neutralizing excess ROS and regulating cellular redox status [51]. This antioxidative property of NO is crucial in preventing cellular damage and maintaining the balance between ROS and RNS signaling under stress conditions, which is essential for proper plant responses. In turn, NO also regulates the expression of antioxidant enzymes, further supporting the plant’s ability to cope with oxidative stress [52].

The interactions between ROS and RNS extend beyond direct molecular interactions, influencing broader epigenetic regulatory mechanisms. Both ROS and RNS are capable of modulating chromatin remodeling and DNA methylation processes, which are essential for the regulation of gene expression in response to environmental stresses. For example, ROS-induced oxidative modifications of chromatin-associated proteins can alter the structure of chromatin, making it more or less accessible for transcriptional activation or repression [53]. Similarly, NO-mediated S-nitrosylation of epigenetic enzymes can influence DNA methylation patterns, thereby regulating gene expression at the epigenetic level [4]. These epigenetic modifications play a crucial role in stress-induced changes in gene expression, enabling plants to mount an adaptive response to various abiotic and biotic stresses.

**Figure 1:**
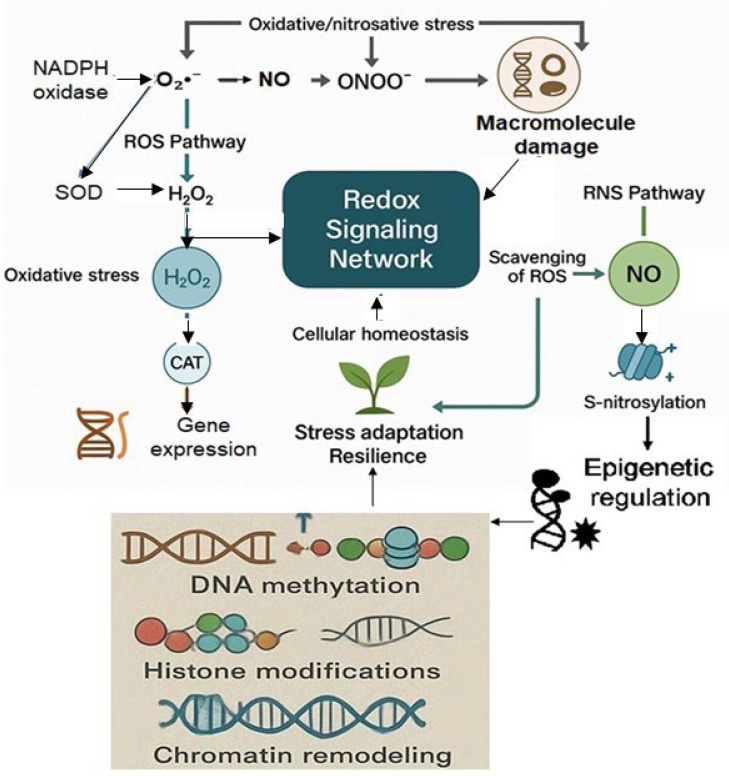
Crosstalk between reactive oxygen species and reactive nitrogen species in plant stress signaling and adaptation. This schematic illustrates the dynamic redox signaling network formed by ROS and RNS under stress conditions. In the ROS pathway, NADPH oxidase-generated superoxide (O_2_•^−^) is converted to hydrogen peroxide (H_2_O_2_) by superoxide dismutase (SOD), which acts as a signaling molecule inducing oxidative stress responses, including gene expression regulation through catalase (CAT) activity. In parallel, nitric oxide (NO) in the RNS pathway contributes to redox balance by scavenging ROS and modulating protein activity through S-nitrosylation. The interaction between O_2_•^−^ and NO produces peroxynitrite (ONOO^−^), a reactive species causing oxidative/nitrosative stress and macromolecular damage. Importantly, both ROS and RNS affect epigenetic regulation by modulating DNA methylation, histone modifications, and chromatin remodeling. H_2_O_2_ can oxidize histone residues, while NO can S-nitrosylate epigenetic enzymes, altering gene expression.

Moreover, the combined action of ROS and RNS in chromatin remodeling and gene expression regulation emphasizes their collaborative role in stress adaptation. The synergistic effects of ROS and RNS are particularly important in improving crop resilience, as the epigenetic changes they induce can lead to the activation of stress-responsive genes and enhance the plant’s ability to tolerate extreme environmental conditions [54,55]. By understanding the molecular mechanisms underpinning ROS and RNS crosstalk, researchers can develop strategies to enhance plant stress tolerance and productivity under unfavorable conditions. As illustrated in Figure 1, the interplay between ROS and RNS forms an integrated redox signaling network that governs plant responses to environmental stress. Their interactions, including peroxynitrite formation and epigenetic regulation, are central to maintaining redox homeostasis and enhancing stress adaptation.

## 3. Epigenetic Regulation by ROS and RNS in Plant Stress Responses

**Figure 2:**
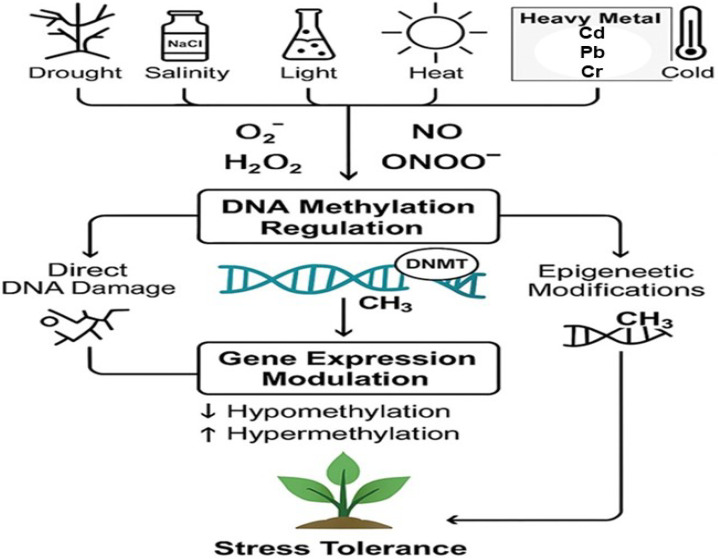
Proposed model of ROS- and RNS-induced DNA methylation modifications in plants under abiotic stress. This schematic illustrates the impact of environmental stressors, such as drought, salinity (NaCl), heat, cold, and excess light, on the generation of reactive oxygen species (ROS) and reactive nitrogen species (RNS) in plant cells. These reactive molecules, including superoxide radicals (O_2_•^−^), hydrogen peroxide (H_2_O_2_), hydroxyl radicals (•OH), nitric oxide (NO), and peroxynitrite (ONOO^−^), influence DNA methylation through two primary mechanisms: direct DNA damage and epigenetic regulation. ROS and RNS interact with DNA methyltransferases (DNMTs), promoting the formation of 5-methylcytosine (5mC) via methyl group (CH_3_) addition to cytosine residues. These modifications modulate gene expression by either activating stress-responsive genes (hypomethylation) or repressing specific gene sets (hypermethylation), ultimately enhancing plant tolerance to abiotic stress conditions.

Epigenetic regulation is a dynamic and heritable mechanism that enables plants to modify gene expression without altering the DNA sequence [56,57]. In the context of abiotic stress, this regulation involves changes in DNA methylation, histone modifications, and small RNA pathways, all of which contribute to stress adaptation [58,59]. Reactive oxygen species and reactive nitrogen species play pivotal roles in modulating these epigenetic processes, acting as signaling molecules that influence chromatin structure and gene accessibility under stress conditions. Figure 2 illustrates the proposed model depicting how ROS and RNS induce DNA methylation modifications in plants subjected to abiotic stress. This section explores the mechanistic details of how ROS and RNS mediate epigenetic regulation in plants.

### 3.1. DNA Methylation and Stress Signaling

DNA methylation is a key epigenetic mechanism in plants that involves the addition of a methyl group to the 5th carbon of cytosine residues, forming 5-methylcytosine (5mC). Unlike animals, where DNA methylation occurs predominantly at CG dinucleotides, plants exhibit methylation in three sequence contexts: CG, CHG, and CHH (where H = A, T, or C) [60,61]. Maintenance of DNA methylation is context-specific: MET1 preserves CG methylation, while CMT3 and CMT2 maintain CHG and CHH methylation, respectively. De novo methylation is established primarily through the RNA-directed DNA methylation (RdDM) pathway, mediated by DRM2 [62,63]. Plants also possess a distinct mechanism for active DNA demethylation, carried out by DNA glycosylases such as ROS1, DME, DML2, and DML3, which remove methylated cytosines via a base excision repair pathway [64,65].

Additionally, active DNA demethylation is catalyzed by DNA glycosylases such as ROS1, DME, and DML2/3, which remove 5mC through a base excision repair mechanism [64,66]. These dynamic processes allow plants to finely tune gene expression, silence transposable elements, and respond adaptively to environmental stresses. Abiotic stresses such as drought, salinity, and heavy metal exposure can induce locus-specific changes in DNA methylation patterns, often mediated by redox signals such as ROS and RNS, ultimately influencing transcriptional reprogramming and stress memory [4,67].

#### 3.1.1. ROS-Induced Modifications in DNA Methylation

ROS, such as H_2_O_2_, O_2_^•−^, and •OH, are known to modulate DNA methylation through several mechanisms. They can directly oxidize DNA bases, leading to modifications like 8-oxoguanine, which influence the binding of DNMTs and demethylases [53,68]. Additionally, ROS act indirectly by triggering signaling cascades that regulate the activity of epigenetic enzymes.

For instance, studies on wounded maize leaves demonstrated that ROS-induced oxidative stress alters DNA methylation patterns rapidly, emphasizing the dynamic and stress-responsive nature of these modifications [69]. In cucumber seedlings, H_2_O_2_ as a primary stress before heat stress resulted in specific DNA methylation changes that protected against growth suppression. This study illustrated the interplay between oxidative signals and DNA methylation machinery, such as DNMTs, which modulate stress-responsive gene expression [70].

ROS-mediated DNA methylation changes are often stress-specific. Drought conditions in wheat lead to the generation of ROS, which influence methylation dynamics, particularly in genes associated with water use efficiency and stress tolerance [71]. Similarly, under salinity stress, pepper cultivars exhibit alterations in DNA methylation, reflecting the role of ROS in driving epigenetic responses tailored to the environmental context [72].

#### 3.1.2. RNS and DNA Methylation

Reactive nitrogen species, particularly NO, contribute to DNA methylation changes by modulating DNMT and demethylase activities. NO can act as a signaling molecule and a regulator of epigenetic enzymes through post-translational modifications like S-nitrosylation. These modifications influence the catalytic activity of DNMTs, altering methylation patterns in stress-responsive genomic regions [73,74].

For example, under heat stress, the application of sodium nitroprusside (SNP), a NO donor, in hyacinth bean plants increased antioxidant enzyme activity and photosynthetic efficiency. This response correlated with DNA methylation changes in stress-regulated genes, highlighting NO’s role as a mediator of epigenetic modifications [74]. NO’s involvement in methylation changes has also been observed in other contexts, such as salinity and drought stress, further underscoring its regulatory potential.

#### 3.1.3. Stress-Specific Modifications in DNA Methylation

Abiotic stress often results in distinct DNA methylation profiles, which are crucial for fine-tuning gene expression and enhancing stress tolerance. Heavy metal exposure induces differentially methylated loci (DML), mediated by ROS and RNS. For instance, manganese (Mn) and cadmium (Cd) stress in pokeweed caused significant shifts in DNA methylation, with ROS acting as key regulators of these changes [75]. In particular, these methylation changes affected promoter regions of genes involved in metal transport, transcriptional regulation, and oxidative stress detoxification. ROS-mediated redox signaling is proposed to either induce DNA demethylation at stress-responsive loci or interfere with methyltransferase activity, thereby facilitating transcriptional reprogramming under metal stress. Having in mind these findings, Shi et al. [76] investigated the effects of excessive copper (Cu) pollution on the physiology of *Hydrilla verticillata*. Their proteomic analysis identified the upregulation of four DNA methylation-related proteins under Cu stress, including homologs of two DRM-type de novo methyltransferases, one CMT-type chromomethylase, and one SUVH6-like histone methyltransferase. Consistently, genome-wide DNA demethylation was observed in Cu-treated plants. Remarkably, treatment with NADPH oxidase inhibitors (DPI and IMZ) restored methylation levels at previously demethylated sites, strongly implicating ROS as a causal factor in Cu-induced DNA hypomethylation. The authors proposed two possible mechanisms: (1) ROS may disrupt methylation by affecting the expression or function of DNA methyltransferases, and (2) ROS-induced DNA damage could hinder the maintenance of methylation during DNA repair. These findings directly link heavy metal-induced redox stress to epigenetic reprogramming in aquatic plants.

Salt stress in kenaf seedlings demonstrated the protective role of methylation inhibitors like 5-azacytidine (5-azaC), which modulate ROS levels and enhance stress tolerance. Pre-treatment with 5-azaC reduced global DNA methylation, as shown by MSAP analysis, and led to increased expression of several stress-responsive genes, including L-ascorbate oxidase (L-AAO). This gene was shown to be functionally required for salt tolerance, as its silencing increased sensitivity to salinity. The demethylation also enhanced antioxidant defenses, boosting activities of superoxide dismutase (SOD), peroxidase (POD), and catalase (CAT), while decreasing superoxide (O_2_^−^) accumulation and lipid peroxidation. This suggests that controlled manipulation of DNA methylation could serve as a strategy to mitigate abiotic stress effects [77]. Furthermore, studies on chickpea genotypes under cold stress revealed genotype-specific DNA methylation patterns influenced by ROS, highlighting the adaptive potential of epigenetic responses [78]. In particular, the cold-tolerant genotype (Sel96Th11439) exhibited progressive DNA demethylation during prolonged cold exposure, especially at loci associated with cold-responsive gene activation. This dynamic methylation shift correlated with enhanced antioxidant enzyme activity—such as SOD, CAT, APX, and GPX—and reduced H_2_O_2_ accumulation, malondialdehyde content, and electrolyte leakage. By contrast, the susceptible genotype (ILC533) showed fewer demethylation events and greater oxidative damage, suggesting that redox-regulated epigenetic flexibility contributes to cold stress resilience in chickpea.

Furthermore, transgenic tobacco plants engineered to overproduce hydrogen peroxide (H_2_O_2_) exhibited genome-wide DNA methylation changes, particularly hypomethylation at CHG sites, which correlated with enhanced resistance to biotic and abiotic stresses [79]. These methylation shifts were associated with differential expression of stress-related genes, including those involved in calcium signaling and energy metabolism, underscoring the role of ROS as a modulator of epigenetic plasticity. Similarly, in rice seedlings exposed to high concentrations of sodium nitroprusside (SNP), a nitric oxide (NO) donor, stress symptoms were accompanied by locus-specific DNA hypomethylation, especially at CHG sites [80]. This epigenetic reprogramming was linked to transcriptional activation of transposable elements and stress-responsive genes, alongside altered expression of chromatin remodeling factors such as *OsCMT3*, *OsDDM1a/b*, and *OsDME*.

Ionizing radiation (IR) represents a significant abiotic stress that induces both direct DNA damage, such as strand breaks and base oxidation, and indirect oxidative stress via ROS accumulation. Chronic gamma irradiation in *Arabidopsis thaliana* across multiple generations leads to widespread CG methylation changes and accumulation of DMRs in genes tied to stress responses, development, and DNA repair, suggesting epigenetic reprogramming may underlie long-term adaptation [81]. Field studies in the Chernobyl and Fukushima Exclusion Zones reveal species-dependent methylation shifts: while *Capsella bursa-pastoris* showed no global change, *Arabidopsis thaliana* exhibited reduced methylation at sites with high radiation dose rates, indicating heritable, environment-specific epigenetic responses [82]. Additionally, rice exposed to heavy-ion radiation shows dose-dependent methylation patterns—low doses promote CG hypermethylation linked to metabolic gene activation, while high doses induce CHG hypomethylation and activate defense pathways [83].

#### 3.1.4. DNA Methylation Dynamics in Plant Stress Adaptation

The dynamic nature of DNA methylation allows plants to respond rapidly to environmental changes. ROS and RNS act as key signaling molecules that initiate epigenetic modifications, ensuring that stress-responsive genes are activated or repressed as needed. In some cases, stress-induced methylation changes are reversible, enabling plants to revert to baseline epigenetic states after stress relief [70,72].

The integration of DNA methylation with other epigenetic mechanisms, such as histone modifications and small RNA regulation, further amplifies its role in plant stress responses. Advances in epigenomic technologies, such as WGBS, have enhanced our ability to study methylation dynamics, providing insights into the molecular basis of stress adaptation [14].

By elucidating the role of ROS and RNS in DNA methylation, researchers can better understand the intricate mechanisms underlying plant resilience to abiotic stress and identify potential targets for genetic or chemical interventions to improve crop performance under adverse conditions.

These studies collectively demonstrate the complex genetic network in which ROS, RNS, and DNA methylation interact to regulate plant stress responses, thus offering information about a possible approach to improve stress resistance in crops and other plant species. A general description of the key studies is given in Table 1.

### 3.2. Histone Modifications and Stress Signaling

Histone modifications play a pivotal role in shaping plant chromatin landscapes and regulating transcriptional responses to environmental stimuli. These covalent post-translational modifications (PTMs) include acetylation, methylation, phosphorylation, ubiquitination, and SUMOylation, primarily on histone H3 and H4 tails [84,85]. In plants, histone acetylation (e.g., H3K9ac, H4K5ac) is generally associated with transcriptional activation, while histone methylation can either activate (e.g., H3K4me3) or repress (e.g., H3K27me3, H3K9me2) gene expression depending on the context [86,87].

These marks are catalyzed by a suite of histone-modifying enzymes: histone acetyltransferases (HATs) such as GCN5, HDACs like HDA6 and HDA19, and histone methyltransferases (HMTs) such as SUVH4/KRYPTONITE, SDG proteins, and PRC2 components [88,89,90].

These modifications impact chromatin structure, making DNA more or less accessible to the transcriptional machinery, thereby modulating gene expression in response to environmental cues [9,91]. Reactive oxygen species and reactive nitrogen species play significant roles in influencing histone modifications, particularly during abiotic stress responses, by modulating the activity of histone-modifying enzymes.

#### 3.2.1. ROS-Induced Histone Modifications

ROS regulate histone modifications both directly and indirectly, primarily through the oxidative regulation of histone-modifying enzymes. Short-term stress often results in reversible histone modifications, while prolonged or severe stress induces more global changes in chromatin structure and histone patterns [9]. For instance, oxidative stress caused by ROS has been shown to increase histone methylation at specific loci, promoting the expression of stress-responsive genes [80,92]. Histone acetylation, typically associated with transcriptional activation, is dynamically regulated by redox signaling during abiotic stress. In maize, heat stress led to increased acetylation of histone marks H3K9, H4K5, and H3, alongside elevated ROS levels and reduced H3K9 di-methylation, contributing to chromatin decondensation and the activation of programmed cell death (PCD) [93]. Conversely, elevated ROS levels may influence histone deacetylation, potentially suppressing gene expression to conserve energy under stress conditions, a mechanism broadly supported in redox-epigenetic literature and discussed in the context of heat stress memory [94].

#### 3.2.2. RNS and Histone Modifications

NS, particularly nitric oxide (NO), regulate histone modifications by inhibiting histone deacetylase (HDAC) activity through *S-nitrosylation*. This redox-dependent post-translational modification reduces HDAC function both in vitro and in vivo, leading to increased histone acetylation at genes involved in stress and defense responses. In *Arabidopsis*, NO donors such as GSNO and salicylic acid-induced NO were shown to trigger this mechanism, highlighting a critical link between NO signaling and chromatin remodeling during stress adaptation [95].

In *Arabidopsis*, NO modulates histone acetylation in a manner dependent on histone deacetylase 6 (HDA6) and S-nitrosoglutathione reductase (GSNOR). NO-sensitive regulation of HDA6 activity results in increased acetylation at histone marks such as H3K9 and H3K9/K14, contributing to a transcriptional shift from growth-related to stress-responsive gene expression [96]. In plants, NO signaling can influence chromatin structure by regulating histone methylation during immune responses. In *Solanum tuberosum* (potato), infection with *Phytophthora infestans* triggers a biphasic NO response, where a transient decline in NO levels at 6 h post-inoculation corresponds with increased expression of defense genes such as *R3a* and *HSR203J* [97]. This expression is associated with the enrichment of the transcriptionally active histone mark H3K4me3 and a reduction in the repressive H4R3 symmetric dimethylation (H4R3sme2) at their promoters. PRMT5, the enzyme catalyzing H4R3sme2, is required for resistance, as its inhibition suppresses defense gene activation and weakens the hypersensitive response. Thus, dynamic NO fluctuations modulate arginine methylation to fine-tune stress-responsive chromatin states in potato immunity.

#### 3.2.3. Crosstalk Between ROS and RNS in Histone Modifications

ROS and RNS often interact to regulate histone modifications synergistically. This crosstalk creates a complex redox signaling network that fine-tunes chromatin accessibility and gene expression under stress. For instance, RNS-mediated S-nitrosylation of histone deacetylases has been shown to regulate histone acetylation and may influence ROS-related pathways by modulating antioxidant gene expression—a concept supported by studies on GSNOR-deficient plants and NO-sensitive chromatin remodeling [11].

**Figure 3:**
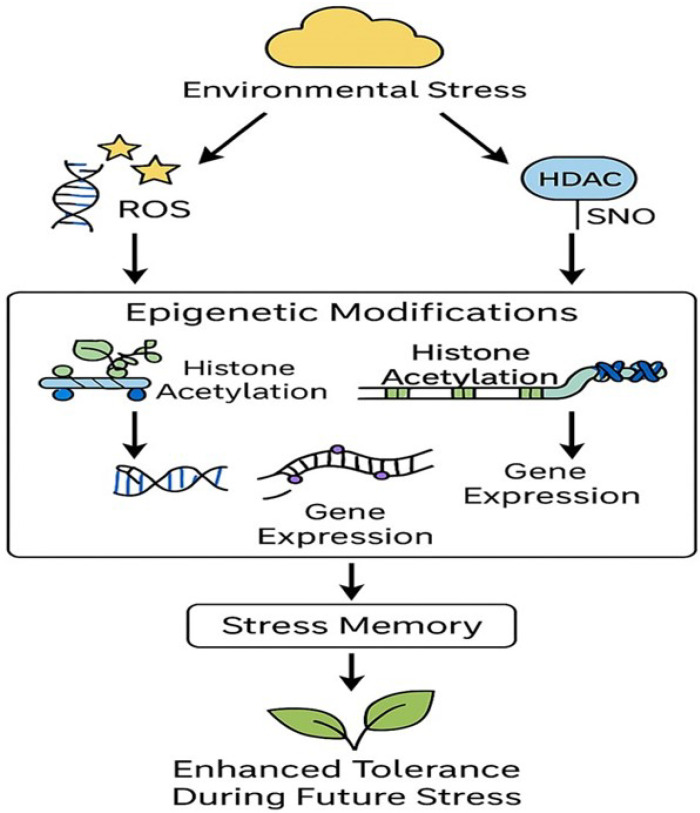
Crosstalk between reactive oxygen species (ROS) and reactive nitrogen species (RNS) in regulating histone modifications and stress memory in plants. This diagram illustrates how environmental stress induces ROS and RNS production in plants, leading to epigenetic modifications of histones. RNS, through S-nitrosylation (SNO) of HDACs, influences histone acetylation, while ROS contributes to changes in chromatin structure and gene expression. These modifications regulate stress-responsive gene activity and contribute to the formation of stress memory. The resulting epigenetic reprogramming enables enhanced plant tolerance during subsequent stress events, supporting long-term adaptation.

Histone modifications are also critical for establishing stress memory, where plants “remember” previous stress encounters and respond more efficiently upon subsequent exposure. For example, salinity stress induces histone methylation patterns in Arabidopsis, which persist after stress removal and enhance tolerance in future stress episodes [91,94]. Figure 3 illustrates the interplay between ROS and RNS in modulating histone modifications under environmental stress. It highlights how ROS and RNS trigger epigenetic changes, including histone acetylation regulated by S-nitrosylation of HDACs, ultimately leading to stress memory and improved plant tolerance during future stress episodes.

#### 3.2.4. Histone Modifications in Specific Stress Responses

Histone modifications play key roles in specific stress responses: under heat stress, increased histone acetylation at specific loci enhances heat tolerance in maize [93] and H_2_O_2_ pretreatment alters histone acetylation in cucumber, protecting against heat-induced damage [70]. Under salt stress, *Arabidopsis* utilizes the histone deacetylase AtSRT2, a sirtuin-like NAD^+^-dependent enzyme, to regulate hydrogen peroxide homeostasis and ensure proper seed germination [98]. AtSRT2 exerts this function by deacetylating histone H4K8 at the promoter of *VAMP714*, a negative regulator of H_2_O_2_-containing vesicle trafficking, thereby repressing its transcription. Loss of AtSRT2 function leads to increased H4K8 acetylation, elevated *VAMP714* expression, higher H_2_O_2_ accumulation, and reduced germination under salt stress. These findings highlight a chromatin-level mechanism that links histone deacetylation to oxidative balance and abiotic stress resilience in early development. Additionally, ADA2b, a transcriptional coactivator in *Arabidopsis*, contributes to abiotic stress responses by modulating histone acetylation and chromatin structure. Specifically, it regulates the expression of stress-inducible genes by controlling H3 and H4 acetylation under salt stress, and influences nucleosome occupancy under low temperature conditions [99]. Using S-nitrosoglutathione (GSNO) as an S-nitrosylating agent and biotin-switch assays followed by mass spectrometry, 135 nuclear proteins were identified as S-nitrosylation targets in *Arabidopsis* after pathogen treatment. Of these, 117 proteins contained at least one cysteine residue and were primarily involved in RNA and protein metabolism, stress response, and cell division. Notably, the identification of two plant-specific HDACs suggests that NO may modulate epigenetic regulation by affecting chromatin structure. These findings provide mechanistic insight into how NO mediates nuclear processes and gene expression during plant defense responses [100]. In support of NO’s role in stress memory, recent findings in potato demonstrate that chemical priming agents, such as β-aminobutyric acid (BABA), γ-aminobutyric acid (GABA), laminarin, and 2,6-dichloroisonicotinic acid (INA), enhance NO synthesis and promote the formation of S-nitrosothiols (SNOs), serving as transient storage forms of NO. This reversible S-nitrosylation, together with NO-triggered upregulation of histone H2B, constitutes a short-term imprinting mechanism that primes the plant for faster and stronger defense responses upon pathogen challenge. The study highlights a dual regulatory mechanism involving redox-based signaling (via SNOs) and epigenetic modifications (e.g., histone alterations), suggesting that finely tuned NO homeostasis is critical for effective immune priming in plants [101].

#### 3.2.5. Histone Modifications and Stress Memory

Histone modifications play a critical role in stress memory, where prior exposure to stress results in epigenetic marks that enhance future responses. ROS and RNS contribute to this memory by modulating histone methylation and acetylation patterns that persist beyond the initial stress event. For example, salinity-induced DNA methylation patterns in pepper plants persist post-stress, enhancing their adaptive capacity to recurring salinity challenges [72].

These stress memory mechanisms involve coordinated regulation of histone-modifying enzymes by ROS and RNS, highlighting the role of redox signaling in long-term stress adaptation.

In the end, these studies sketch a quite clear picture of how ROS, RNS signaling pathways, and particularly NO affect histone modifications. This process of stress signals interacting with epigenetic regulation enables plants and other organisms to cope with a variety of environmental issues.

Table 2 provides a comprehensive overview of some important studies that have investigated the interaction of oxidative stress and nitric oxide with histone epigenetic regulation in plants.

### 3.3. Small RNA Regulation and Plant Stress Responses

Small RNAs (sRNAs) are short, non-coding RNA molecules (20–24 nt) that regulate gene expression in plants at both the transcriptional and post-transcriptional levels [102,103]. The two major classes are microRNAs (miRNAs) and small interfering RNAs (siRNAs), each playing distinct roles in stress adaptation and developmental processes [104].

miRNAs are processed from endogenous transcripts with imperfect self-complementarity and typically guide post-transcriptional gene silencing (PTGS) by directing mRNA cleavage or translational repression via AGO1 [105,106]. In contrast, 24-nt siRNAs, especially those derived from transposons and repeats, function via the RNA-directed DNA methylation (RdDM) pathway. This plant-specific mechanism involves RNA Polymerase IV and V, Dicer-like 3 (DCL3), Argonaute 4 (AGO4), and Domains Rearranged Methyltransferase 2 (DRM2) to guide cytosine methylation at homologous loci, resulting in transcriptional gene silencing (TGS) [107,108,109].

The plants are observed to have a very complicated defense mechanism to deal with many and different environmental stressors. This system uses a very complex mechanism that combines signaling pathways and gene regulation. Regulation of genes at the post-transcriptional level one of the key roles of small RNAs, and this is especially true of miRNAs [110,111].

#### 3.3.1. miRNAs and ROS Signaling

Consequently, the ROS produced from stress can affect miRNA expression and function. For example, the research team of Wang et al. [13] uncovered taemiR9674a in wheat that improves stress resilience by controlling the expression of genes for osmotic stress defenses and ROS equilibrium. Mechanistically, the miRNA is shown to target seven genes, which are implicated in growth traits of plants under stress. In addition to growth promotion and tolerance improvement, tobacco overexpression of *taemir9674a* contributed to the biomass.

This upregulation, in turn, facilitates the development of antioxidant enzymes and thus the maintenance of redox homeostasis and osmotic stress responses. Transcriptome analysis has been proved to regulate genes that are associated with phytohormones, salt response, and ROS homeostasis, demonstrating its crucial role in osmotic stress tolerance. In wheat, virus-derived small interfering RNA (vsiRNA1) enhances resistance to multiple pathogens by modulating ROS homeostasis [112]. vsiRNA1 targets and suppresses *TaAAED1*, a thioredoxin-like gene that normally dampens chloroplast ROS production. This derepression of ROS promotes antiviral defense, highlighting a small RNA-based redox regulatory mechanism in immune signaling.

In parallel, plant microRNAs (miRNAs) function as key post-transcriptional regulators under abiotic stress by targeting transcripts involved in redox balance, DNA repair, and hormone signaling. Exposure to stressors such as drought, salinity, temperature extremes, and heavy metals can damage both nuclear and organelle DNA, triggering miRNA-mediated regulatory responses. By modulating gene networks associated with genome stability and oxidative stress, miRNAs help coordinate adaptive signaling pathways that preserve cellular homeostasis under adverse environmental conditions [113]. Similarly, under biotic stress, miR398b enhances rice immunity against *Magnaporthe oryzae* by regulating superoxide dismutase genes, leading to increased hydrogen peroxide (H_2_O_2_) accumulation. This underscores the role of miRNAs in redox-based defense signaling during pathogen attack [114].

In the model grass *Brachypodium distachyon*, oxidative stress imposed by H_2_O_2_ triggers a distinct small RNA response. High-throughput sequencing identified 31 known and 30 novel H_2_O_2_-responsive miRNAs out of 144 known and 221 candidate new miRNAs. RT-qPCR and 5′-RACE validation showed that several of these miRNAs cleave or repress target transcripts involved in ROS detoxification and stress-signaling pathways, thereby constructing a miRNA-mediated regulatory network that fine-tunes the oxidative-stress defense response [115].

**Figure 4:**
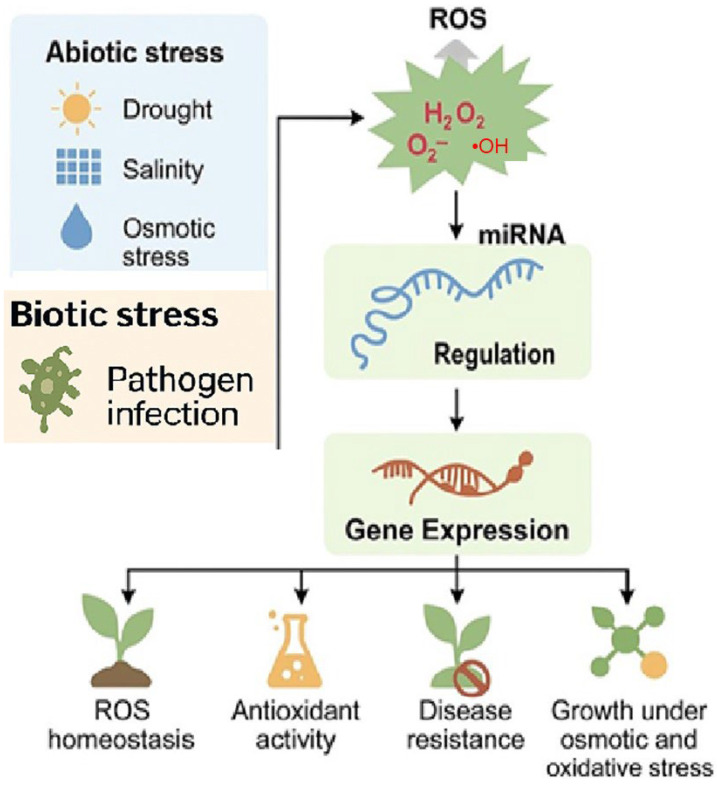
Proposed model of ROS-mediated miRNA regulation and gene expression in plant stress tolerance. This model illustrates how abiotic stresses, including drought, salinity, and osmotic stress, as well as biotic stresses such as pathogen infection induce the generation of reactive oxygen species (ROS) such as hydrogen peroxide (H_2_O_2_), superoxide (O_2_^−^), and hydroxyl radicals (•OH). These ROS function as signaling molecules that modulate the expression of specific microRNAs (miRNAs). The miRNAs, in turn, regulate gene expression involved in redox balance, antioxidant enzyme activity, pathogen defense, and growth under stress. This post-transcriptional regulation supports ROS homeostasis, enhances antioxidant defenses, improves disease resistance, and promotes adaptation to oxidative and osmotic stress.

Expression patterns were verified by RT-qPCR and target genes were verified experimentally, thus the regulation mechanism of miRNA in post-transcriptional response to oxidative stress and defense was elucidated. This can be used to explain the correlation between H_2_O_2_ stress and miRNA regulation. These studies demonstrate an important role of miRNAs in this signaling pathway under stress conditions. Figure 4 presents a model depicting how ROS generated under abiotic stress conditions modulate specific miRNAs, which in turn regulate genes associated with redox homeostasis, antioxidant defense, and stress adaptation.

#### 3.3.2. miRNAs and NO

**Figure 5:**
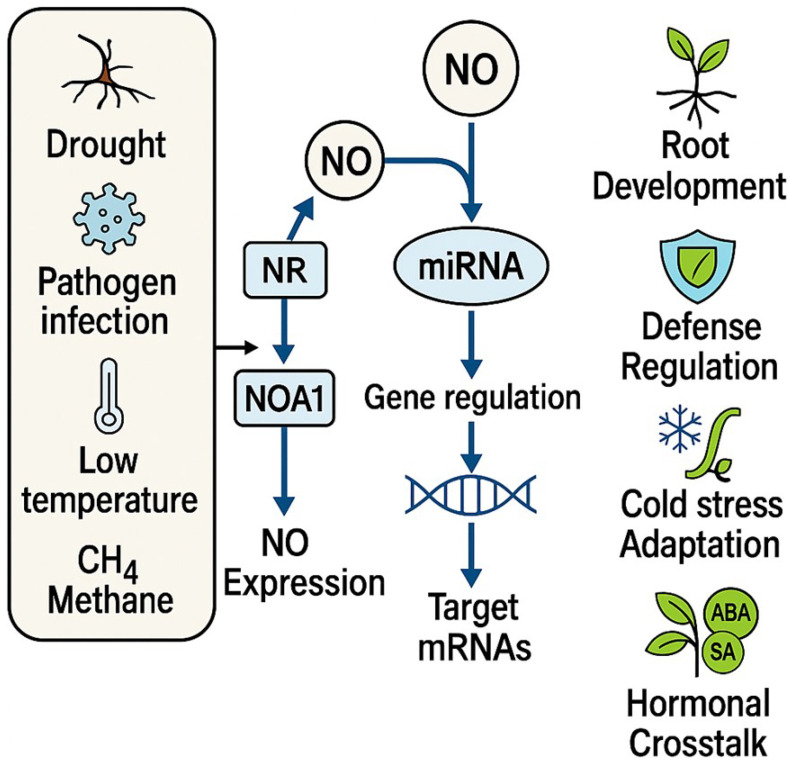
Proposed model of NO-mediated miRNA regulation in plant stress responses and development. Abiotic (drought, low temperature, methane) and biotic (pathogen infection) stresses activate NO biosynthesis through nitrate reductase (NR) and NO-associated protein 1 (NOA1). The resulting NO modulates microRNA (miRNA) expression, which in turn targets specific mRNAs. These mRNAs encode key regulators of stress adaptation, contributing to enhanced root development, defense mechanisms, cold stress tolerance, and hormonal crosstalk (including abscisic acid [ABA] and salicylic acid [SA] pathways).

Nitric oxide is also identified as one of the critical players in stress reactions of plants. As Singh et al. [116] pointed out, the crosstalk between miRNA and NO signaling is a highly important aspect of response to stress. Zhao et al. [117] reported that NO amendment does not alter the expression of specific miRNA in alfalfa under drought stress. Next-generation sequencing revealed the presence of 90 known miRNAs and made a prediction of 177 new miRNAs. The fact that there are differentially expressed miRNAs affected by NO treatment suggests that NO has some regulating effect on miRNA. NO analysis in the context of target genes reveals the mechanisms behind drought response. RT-qPCR validation confirms that NO induces miRNAs targeting genes essential for drought tolerance, therfore NO could be used to improve alfalfa stress tolerance through miRNA-mediated regulatory pathways. Jin et al. [118] revealed that methane (CH_4_) promotes lateral root formation in tomato and *Arabidopsis* by triggering NO production in root tissues. This CH_4_-induced NO acts as a signaling molecule that upregulates key cell cycle genes (*SlCYCA2;1*, *SlCYCA3;1*, *SlCDKA1*) and downregulates the cell cycle inhibitor *SlKRP2*. Simultaneously, NO modulates the expression of root development-related microRNAs (*SlmiR160*, *SlmiR390a*) and their targets (*SlARF16*, *SlARF4*), coordinating root architecture remodeling. Scavenging NO abolished these effects, confirming its essential role as a downstream signal in CH_4_-mediated root development. In black pepper (*Piper nigrum*), infection by *Phytophthora capsici* activates NO biosynthesis as part of the defense response. While the nitrate reductase gene (*Pn1_NR*) is upregulated, the NO-associated gene (*Pn1_NOA1*) is paradoxically downregulated, a regulation shown to result from miRNA-mediated cleavage of *Pn1_NOA1* mRNA. This post-transcriptional silencing was supported by sRNAome analysis and validated through modified 5′ RLM-RACE. These findings reveal a layered defense system in which NO signaling and miRNA networks intersect to fine-tune stress responses in black pepper [119]. In addition, Xu et al. [120] demonstrated that low temperature (LT) and NO coordinately regulate *Camellia sinensis* pollen tube growth through miRNA-mediated pathways. Low temperature and NO each induce distinct sets of differentially expressed miRNAs (DEMs), which target genes involved in cytoskeletal dynamics, redox regulation, and calcium signaling. Specifically, NO-responsive miRNAs modulate genes related to redox balance and Ca^2+^ gradient maintenance, ultimately influencing polar tip growth under cold stress. Ruan et al. [121] demonstrated that endogenous nitric oxide (NO) enhances drought tolerance in alfalfa by modulating hormone signaling and regulating stress-related miRNAs. NO influenced the levels of key stress hormones, particularly abscisic acid (ABA) and salicylic acid (SA), and induced NO-responsive miRNAs enriched in pathways related to hormone signaling, phenylpropanoid metabolism, and stress adaptation. These findings underscore the coordinated role of NO and miRNAs in shaping drought responses at both physiological and molecular levels. As illustrated in Figure 5, NO functions as a key signaling molecule that modulates miRNA expression in response to various environmental stressors. The resulting NO-induced miRNAs regulate specific target genes involved in root development, defense responses, cold stress adaptation, and hormonal signaling, highlighting the central role of the NO–miRNA network in plant stress resilience.

Finally, NO plays a crucial role in plant stress responses by intricately interacting with miRNAs to regulate gene expression under diverse environmental stresses. NO modulates miRNA expression, influencing pathways crucial for stress adaptation and plant development. This interplay involves NO-mediated changes in miRNA profiles targeting genes involved in redox balance, hormone signaling, and stress responses, underscoring NO’s pivotal role as a signaling molecule in plant adaptation mechanisms.

The crosstalk among microRNAs and signaling molecules like ROS and RNS, mainly nitric oxide, is critical for plant stress responses (Table 3).

## 4. Recent Advances in Epigenomic Technologies for Studying ROS and RNS-Mediated Epigenetic Regulation

Recent progress in epigenomic technologies has significantly advanced understanding of how ROS and RNS mediate epigenetic modifications in plants exposed to biotic and abiotic stresses. These tools allow high-resolution profiling of DNA methylation, histone modifications, and non-coding RNA dynamics, offering comprehensive insights into redox-regulated chromatin remodeling and transcriptional reprogramming. This section highlights major advances in epigenomic methodologies and their application in elucidating the role of ROS and RNS in plant stress epigenetics.

### 4.1. Whole-Genome Bisulfite Sequencing (WGBS)

Whole-genome bisulfite sequencing is a powerful high-throughput technique that enables single-base resolution mapping of 5-methylcytosine (5mC) across the entire genome [121,122]. This technology has revolutionized the study of plant stress epigenetics by enabling detailed analysis of context-specific DNA methylation patterns (CG, CHG, and CHH contexts) under varying environmental conditions.

WGBS has revealed that ROS and RNS modulate DNA methylation by affecting the activity of DNMTs and demethylases. For instance, under drought stress, WGBS analysis in *Morus alba* demonstrated ROS-induced differential methylation in promoter regions of genes involved in water-use efficiency and osmotic regulation [14]. Similarly, in wheat, drought-triggered ROS accumulation led to distinct differentially methylated regions (DMRs) associated with transcriptional changes in stress-responsive loci [71].

These studies underscore WGBS as a critical tool in identifying epigenetic signatures that mediate adaptive responses to ROS- and RNS-associated stress signaling.

### 4.2. Chromatin Immunoprecipitation Sequencing (ChIP-Seq)

Chromatin immunoprecipitation followed by sequencing (ChIP-seq) allows for genome-wide identification of protein–DNA interactions, including histone modifications and transcription factor (TF) binding. This method has become essential for delineating chromatin states and epigenetic landscapes under stress conditions [123].

ChIP-seq enables genome-wide mapping of key histone modifications such as H3K9ac, H3K4me3, H3K27me3, and H3K36me2, which influence chromatin accessibility and transcriptional regulation. For example, under cold stress in maize, ChIP-seq analysis revealed genome-wide changes in H3K9 acetylation (H3K9ac), with notable reductions particularly across gene-rich regions, linking histone acetylation dynamics to cold acclimation [124].

Despite initial technical hurdles in plant systems, such as rigid cell walls, heterogeneous tissue composition, and low abundance of nuclear proteins, protocol improvements have significantly enhanced the efficiency of chromatin isolation and immunoprecipitation. Innovations including formaldehyde crosslinking optimization, MNase digestion, and tissue-specific enrichment have extended ChIP-seq utility to both model and non-model species [125,126,127].

Importantly, ChIP-seq has elucidated how redox signals influence histone-modifying enzymes. For instance, in *Arabidopsis thaliana*, nitric oxide-mediated S-nitrosylation of HDA19, a histone deacetylase, was shown to impact histone acetylation patterns under oxidative stress, as revealed by ChIP-seq [15]. These findings highlight a mechanistic link between redox signaling and histone modification, mediated by post-translational regulation of chromatin remodelers.

### 4.3. Small RNA Sequencing

Small RNA sequencing (sRNA-seq) is a high-throughput approach designed to capture and quantify microRNAs (miRNAs), small interfering RNAs (siRNAs), and other small non-coding RNAs, many of which play critical roles in transcriptional and post-transcriptional regulation during abiotic stress responses [128].

sRNA-seq has been instrumental in identifying stress-responsive small RNAs that are regulated by ROS and RNS signaling pathways. For example, miRNAs targeting antioxidant enzymes and signaling proteins are differentially expressed in response to ROS accumulation under drought and salinity stress [129,130]. Moreover, ROS- and RNS-regulated siRNAs have been shown to guide DNA methylation and gene silencing at stress-associated loci through the RNA-directed DNA methylation (RdDM) pathway [131,132].

**Figure 6:**
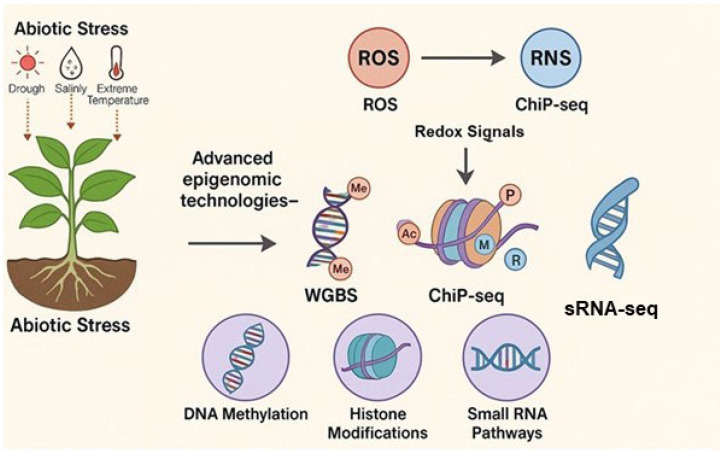
Technological platforms linking redox signaling to epigenetic regulation under abiotic stress. Abiotic stresses such as drought, salinity, and extreme temperatures trigger the production of ROS and RNS, which in turn modulate key epigenetic mechanisms. This figure illustrates how epigenomic platforms, including whole-genome bisulfite sequencing (WGBS), chromatin immunoprecipitation sequencing (ChIP-seq), and small RNA sequencing (sRNA-seq), enable high-resolution mapping of stress-induced modifications in DNA methylation, histone marks, and small RNA pathways. These technologies provide a foundational framework to decode the redox-sensitive epigenetic landscape that shapes plant stress responses and chromatin dynamics.

**Figure 7:**
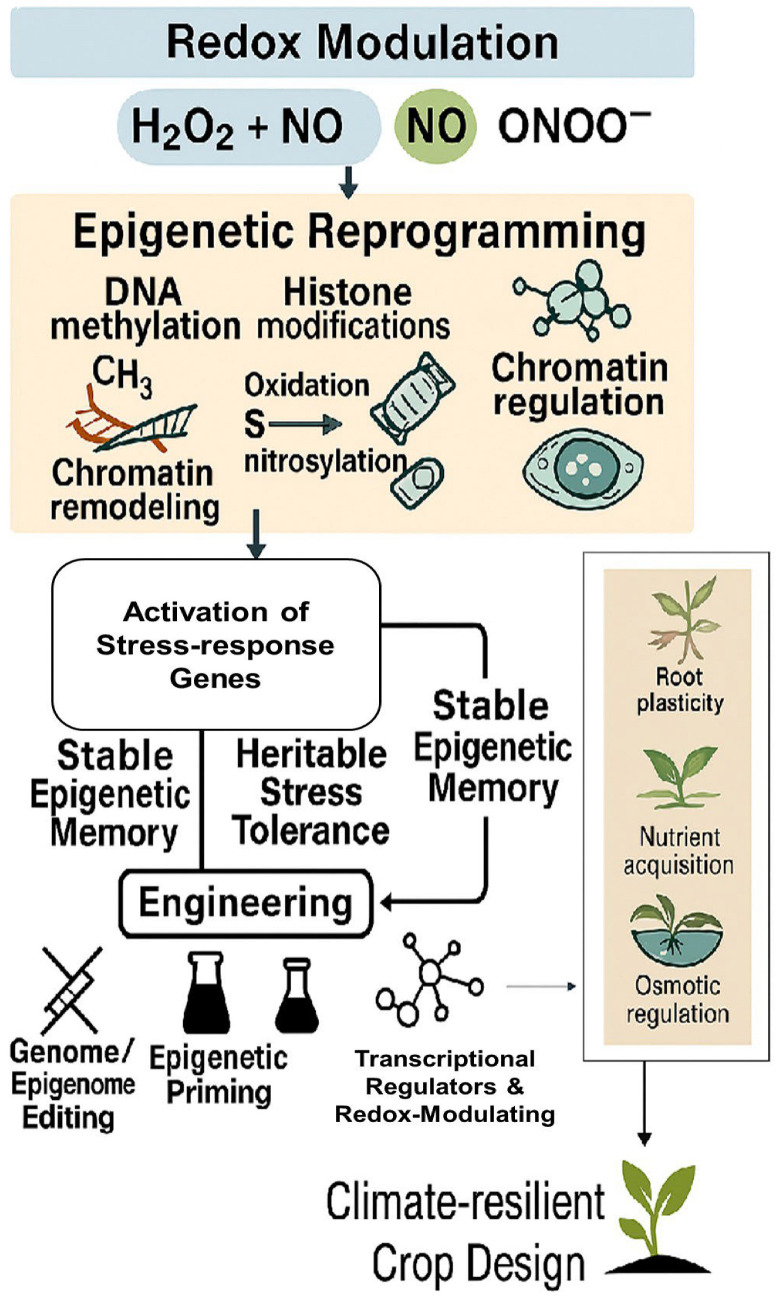
Integrative framework linking redox signaling and epigenetic modulation to stress-resilient crop engineering. Reactive oxygen species (e.g., H_2_O_2_) and reactive nitrogen species (e.g., NO, ONOO^−^) act as redox signals that trigger epigenetic reprogramming through mechanisms such as DNA methylation, histone modifications (e.g., acetylation, methylation), chromatin remodeling, and chromatin regulation. These modifications collectively activate stress-response genes, contributing to stable epigenetic memory and heritable stress tolerance. Through targeted engineering strategies, such as genome/epigenome editing, epigenetic priming, and modulation of transcriptional regulators, these memory traits can be enhanced to improve root plasticity, nutrient acquisition, and osmotic regulation. Together, these processes support the development of climate-resilient crop varieties.

In addition to profiling known small RNAs, sRNA-seq, when paired with degradome sequencing, enables de novo discovery of novel regulatory RNAs and validation of their target transcripts [133]. These technological platforms (Figure 6) lay the analytical foundation for targeted crop improvement. Building on this, Figure 7 presents an application-driven framework that integrates redox biology with epigenetic engineering to guide climate-resilient crop design.

Collectively, the integration of WGBS, ChIP-seq, and small RNA sequencing offers a multi-dimensional view of ROS- and RNS-mediated epigenetic regulation in plants. These technologies not only map molecular changes in the epigenome but also uncover the underlying redox-sensitive mechanisms that coordinate gene expression during stress adaptation. Continued technological advances and interdisciplinary approaches are expected to drive deeper insights into epigenetic signaling networks and facilitate the engineering of stress-resilient crops.

## 5. Concluding Remarks and Future Directions

Plants are constantly challenged by diverse environmental stresses, and the intersection of redox biology and epigenetic regulation represents a powerful axis for understanding how they perceive, respond to, and even remember such challenges. Reactive oxygen and nitrogen species (ROS and RNS) are no longer viewed merely as metabolic byproducts or damage signals; they serve as key modulators of DNA methylation, histone modifications, and small RNA pathways, reprogramming chromatin landscapes to enable dynamic and heritable stress adaptation.

Advances in sequencing technologies, such as whole-genome bisulfite sequencing (WGBS), chromatin immunoprecipitation sequencing (ChIP-seq), and small RNA sequencing, have helped illuminate these interactions at the genome-wide scale. Yet a precise picture of how redox cues are transduced into lasting epigenetic states across tissues and stress types remains incomplete, particularly under complex or simultaneous stress conditions.

Future research must embrace real-time biosensors in combination with high-resolution epigenomic platforms to capture dynamic redox-epigenetic changes in vivo. Clarifying the redox regulation of key chromatin modifiers, such as methyltransferases, demethylases, and acetyltransferases like GCN5/ADA2b, will be essential to decipher the molecular basis of stress memory and priming. The persistence of stress-induced epigenetic marks across generations opens possibilities for non-transgenic crop enhancement.

Innovative strategies such as CRISPR/dCas9-driven epigenome editing, targeted to stress-responsive loci and coupled with ROS/RNS priming, offer unprecedented precision in tuning gene expression for improved resilience. Translating these laboratory insights into the field will require integrative systems biology models that connect redox dynamics with epigenomic, transcriptomic, proteomic, and phenotypic layers, laying the foundation for predictive agriculture and climate-resilient cultivar design.

In sum, redox signaling and epigenetic plasticity stand at the heart of how plants respond to an ever-changing world. By harnessing these intertwined pathways, we can move beyond traditional stress tolerance strategies and toward a future of smart, adaptive crops engineered for resilience and sustainability.

## Figures and Tables

**Table 1:** Overview of studies investigating the relationship between oxidative stress, nitric oxide, and DNA methylation in plants.

Stress Type	Species/Plant Model	Main Findings	Genes/Proteins Involved	Reference
Heat + H_2_O_2_	*Cucumis sativus* (Cucumber)	Exogenous H_2_O_2_ modulated heat-induced DNA methylation; altered expression of methylation at specific loci and mitigated growth suppression	*Csa026131*, *Csa012834*, *Csa015520*	[70]
Salinity	*Capsicum annuum* (Pepper)	Salinity altered methylation in a cultivar-dependent manner; demethylation linked to salt tolerance in ‘Maras’ cultivar	Not specified	[72]
Drought + NO	*Dendrobium huoshanense*	Exogenous NO via SNP reduced methylation and increased antioxidant enzyme activity under drought stress	Not specified	[73]
Heat + SA/NO	*Lablab purpureus* (Hyacinth bean)	SA and SNP modulate DNA methylation patterns under high temperature; correlated with improved physiological traits	Not specified	[74]
Heavy Metals (Mn, Cd)	*Phytolacca americana* (Pokeweed)	HM-induced ROS modulate DNA methylation; DMLs were stress- and ROS-dependent	*MET1*, *CMT2*, *CMT3*, *ROS1*, *RBOH*	[75]
Heavy Metals (Cu)	*Hydrilla verticillata*	Cu-induced ROS affected DNA methylation; ROS inhibition reversed demethylation	*DRM*, *CMT*, *SUVH6*, *ROS1*, *RBOH*	[76]
Salinity + DNA demethylation inhibitor	*Hibiscus cannabinus* (Kenaf)	5-azaC pretreatment reduced methylation and improved stress tolerance by altering gene expression and ROS levels	*L-AAO* (virus-induced silencing increased sensitivity)	[77]
Cold Stress	*Cicer arietinum* (Chickpea)	Cold-tolerant genotype showed higher methylation and antioxidant response; prolonged stress enhanced demethylation for gene activation	Not specified	[78]
Oxidative Stress (endogenous H_2_O_2_)	Transgenic *Nicotiana tabacum* (Tobacco)	High endogenous H_2_O_2_ altered CHG methylation; 9432 DMRs, with functional links to respiration and Ca^2+^ signaling	83 DEGs affected by DMRs	[79]
NO toxicity	*Oryza sativa* (Rice)	High SNP caused CHG hypomethylation and gene/TE activation; altered chromatin regulators	*OsCMT3*, *OsDDM1a*, *OsDDM1b*, *OsDME*	[80]
Ionizing Radiation (IR)	*Arabidopsis thaliana*	Multi-generational IR exposure led to cumulative DMRs; CG methylation was most affected; many DMRs associated with stress/development genes	Not specified	[81]
Environmental Radiation	*A. thaliana* and *Capsella bursa-pastoris*	A. thaliana in Chernobyl showed reduced global methylation; Capsella in Fukushima showed no change	Not specified	[82]
Heavy-Ion Radiation (HIR)	*Oryza sativa* (Rice)	Dose-dependent methylation patterns: low dose hypermethylation (CG), high dose hypomethylation (CG); CHG hypomethylation occurred in both	Not specified	[83]

**Table 2:** Redox and nitric oxide-mediated regulation of histone modifications in plant stress responses.

Study	Key Findings	Implications	Key Genes/Proteins	Reference
*Arabidopsis thaliana*—HDA19 S-nitrosylation	Nitric oxide-dependent S-nitrosylation of four cysteines (Cys137 critical) enhances HDA19 nuclear enrichment, target binding, histone deacetylation, and repression of stress genes. Loss of HDA19 disturbs redox balance and stress tolerance.	Demonstrates redox sensing at the chromatin level via post-translational control of an HDAC.	*HDA19*	[15]
*Zea mays* —heat stress	Heat triggers ROS accumulation, acetyl-H3K9/H4K5/H3, H3K9me2, chromatin decondensation, and programmed cell death (PCD). Trichostatin A mimics hyper-acetylation and PCD.	Links ROS-driven histone acetylation changes to PCD in leaves.	*SOD*, *CAT*, *POD*	[93]
*Arabidopsis thaliana*—NO and HDAC activity	GSNO inhibits total HDAC activity; genome-wide H3K9/14ac hyper-acetylation at defense genes. SA elevates NO, reproducing effect.	NO acts upstream of HDACs to activate stress-responsive transcription.	*HDA6*, H3K9/14ac loci	[95]
*Arabidopsis thaliana*—light/NO/HDA6	Light-dependent NO shifts global H3/H3K9/K9-14 acetylation via NO-sensitive *HDA6*; requires *GSNOR*.	Connects environmental light cues, NO, and chromatin to reprogram metabolism toward stress defense.	*HDA6*, *GSNOR*	[96]
*Solanum tuberosum*—pathogen/PRMT5	Decline in NO at 6 hpi coincides with H3K4me3 and H4R3sme2 at defense promoters (*R3a*, *HSR203J*); *PRMT5* inhibition blocks resistance.	NO dynamics and arginine methylation coordinate late-blight immunity.	*PRMT5*, *R3a*, *HSR203J*	[97]
*Arabidopsis thaliana*—*AtSRT2* and salt	NAD^+^-dependent HDAC *AtSRT2* deacetylates H4K8 at *VAMP714* promoter; loss of *AtSRT2*, H4K8ac, *VAMP714*, H_2_O_2_, and germination under salt stress.	Shows HDAC-controlled redox homeostasis during seed germination.	*AtSRT2*, *VAMP714*	[98]
*Arabidopsis thaliana*—nuclear S-nitrosylome	135 nuclear proteins S-nitrosylated after pathogen; includes two plant-specific HDACs, as well as numerous transcription and RNA-processing factors.	Expands NO target list; supports NO regulation of nuclear epigenetic machinery.	Multiple HDACs, nuclear regulators	[100]
*Solanum tuberosum*—chemical priming/NO	Priming agents (BABA, GABA, laminarin, INA) raise NO and reversible S-nitrosothiols (SNOs); together with *H2B* upregulation create a short-term “imprint” for faster defense.	Highlights NO-SNO-histone axis underlying defense priming.	*H2B*, SNO storage proteins	[101]

**Table 3:** ROS and RNS-mediated regulation of miRNA expression and gene silencing in plant stress responses.

Stress Condition	miRNA Function	Signaling Molecule(s)	Key Genes/Proteins	Reference
Drought and Salt (*Triticum aestivum*, *Nicotiana tabacum*)	taemiR9674a regulates osmotic stress response, ROS homeostasis, and growth traits	ROS	*NtP5CS1*, *NtFeSOD*, *NtCAT1*, *NtPOD4*	[13]
Viral infection (*Triticum aestivum*)	vsiRNA1 enhances virus resistance by silencing negative ROS regulator	ROS	*TaAAED1*	[112]
Fungal pathogen (*Oryza sativa*)	miR398b enhances resistance via SOD gene regulation and H_2_O_2_ production	ROS	*CSD1*, *CSD2*, *SODX*, *CCSD*	[114]
Oxidative stress (H_2_O_2_, *Brachypodium distachyon*)	Novel/conserved miRNAs regulate ROS-responsive genes	ROS	*Bradi2g53010*, *Bradi1g36540*, *Bradi2g52840*, *Bradi2g55497*, *Bradi4g01380*, *Bradi1g11800*, *Bradi3g57320*, *Bradi4g08140*	[115]
Methane-induced root growth (*Solanum lycopersicum*, *Arabidopsis thaliana*)	miR160 and miR390a mediate CH_4_-induced lateral root development via NO signaling	RNS	*SlARF16*, *SlARF4*, *SlCYCA2;1*, *SlCYCA3;1*, *SlCDKA1*, *SlKRP2*	[118]
Pathogen stress (*Piper nigrum*)	miRNA-mediated cleavage of *NOA1* mRNA affects NO biosynthesis	RNS	*Pn1_NR*, *Pn1_NOA1*, *Pn1_NOA2*	[119]
Cold stress (*Camellia sinensis*)	NO-regulated miRNAs modulate redox genes, Ca^2+^ signaling, and cytoskeleton remodeling	RNS	Redox-, metal ion-, actin-, and cell wall-related genes	[120]
Drought (*Medicago sativa*)	NO-responsive miRNAs regulate hormone signaling to improve drought tolerance	RNS	ABA, SA, ETH, and JA pathway-related genes	[121]
